# Extended List of Warning Signs in Qualification to Diagnosis and Treatment of Inborn Errors of Immunity in Children and Young Adults

**DOI:** 10.3390/jcm12103401

**Published:** 2023-05-11

**Authors:** Anna Dąbrowska, Elżbieta Grześk, Anna Urbańczyk, Marta Mazalon, Grzegorz Grześk, Jan Styczyński, Sylwia Kołtan

**Affiliations:** 1 Department of Pediatrics, Hematology and Oncology, Faculty of Medicine, Ludwik Rydygier Collegium Medicum in Bydgoszcz, Nicolaus Copernicus University, 9 Skłodowskiej-Curie St., 85-094 Bydgoszcz, Poland; anna.dabrowska@cm.umk.pl (A.D.); aurbanczyk@cm.umk.pl (A.U.);; 2 Department of Cardiology and Clinical Pharmacology, Faculty of Health Sciences, Ludwik Rydygier Collegium Medicum in Bydgoszcz, Nicolaus Copernicus University, 75 Ujejskiego St., 85-168 Bydgoszcz, Poland; g.grzesk@cm.umk.pl

**Keywords:** inborn errors of immunity, primary immunodeficiency diseases, warning signs

## Abstract

Background and Objectives: Inborn errors of immunity (IEI) refer to genetically determined disorders presenting with recurrent infections, autoimmunity, allergies, and malignancies. IEI is now commonly used, replacing the previously used term primary immunodeficiencies (PID). The 10 warning signs of IEI are widely used in the identification patients with IEI. The aim of the study was to determine and compare the utility of the 10 and 14 warning signs in IEI diagnosing. Methods: A retrospective analysis of 2851 patients was performed (98.17% were subjects under 18 years old and 1.83% were adults). All patients were questioned about the 10 warning signs and four additional signs: severe eczema, allergies, hemato-oncologic disorders and autoimmunity. Sensitivity, specificity, positive and negative predictive values, and odds ratio were calculated for the 10 and 14 warning signs. Results: IEI were diagnosed in a total of 896 (31.4%) patients and excluded in 1955 (68.6%). The strongest predictors of IEI were hemato-oncologic disorders (OR = 11.25; *p* < 0.001) and autoimmunity (OR = 7.74; *p* < 0.001). The strongest predictors of severe IEI were hemato-oncologic disorders (OR = 89.26; *p* < 0.001), positive family history (OR = 25.23; *p* < 0.001), and autoimmunity (OR = 16.89; *p* < 0.001). There were 20.4% and 14% of IEI patients without any signs from the 10 and 14 warnings signs, respectively (*p* < 0.001). 20.3% and 6.8% of patients with severe PIDs had no presence of any signs from 10 and 14 signs, respectively (*p* = 0.012). Conclusions: The 10 warning signs have limited usefulness in identifying IEI. The modified list of 14 warning signs seems to represent an effective diagnostic method for the detection of IEI patients, especially those with severe PIDs.

## 1. Introduction

Inborn errors of immunity (IEI) are a group of heterogeneous, inherited disorders that rely on defects of morphology, origin and/or function of the immune system [1,2]. IEI have a wide spectrum of clinical presentations, involving not only infections, but also auto-immunization, allergies, neoplasms, and lymphoid proliferation [3,4,5,6,7,8,9]. IEI include, but are not only limited to, primary immunodeficiency disorders (PID). In the last decade there has been a rapid increase in the rate of discovery of new genetic defects in PIDs, largely due to the advent of next generation sequencing. This is why, since 2019, there has been a move to name these conditions from PID to IEI. As of 2020, there are more than 480 IEI. IEI is now commonly used, replacing the previously used term primary immunodeficiencies (PID) [10,11].

The early diagnosis of IEI is essential for survival and reducing healthcare costs [12,13,14,15]. It has been estimated that 65% of children with IEI need their first medical help from the family doctor [16].

In 1987, Vicki and Fred Modell founded the Jeffrey Modell Foundation (JMF) in memory of their son Jeffrey, who died due to IEI. The Jeffrey Modell Foundation, in collaboration with immunologists, has compiled a list of 10 warning symptoms of IEI (Table 1). The role of the JMF warning signs was to promote awareness of IEI among physicians, especially general practitioners who can consider IEI when two or more warning signs are present [17,18].

More recent observations indicate that the JMF warning signs are not ideal [18,19] due to one third of patients with IEI not fulfilling any criteria from the list [16] and some symptoms such as failure to thrive are not adapted to adults [20]. In the case of a first child with IEI in a family or some severe IEI, the 10 warning signs may not be useful [16].

Several proposals for variants of the JMF list have been published in the literature. One of them is a list of 12 warning signs of IEI in infants, including in addition to infectious symptoms, inter alia, disseminated Bacillus Calmette-Guerin (BCG) infection after vaccination against tuberculosis, congenital heart defects, hypocalcaemia, and autoimmune symptoms. Despite the broad spectrum of symptoms of the list, its actual diagnostic usefulness has not been assessed [21]. On the other hand, for adults, ESID proposed the 6 ESID warning signs for adult primary immunodeficiency diseases, differing from the JMF list only in the type and time of infection [22]. The list also does not take into account the non-infectious symptoms of IEI. Arslan et al. pointed out that no consideration to the non-infectious symptoms of IEI in the six ESID warning signs greatly excludes patients with IEI [23]. Eldeniz et al. also emphasized the need for modification of the 10 warning signs of IEI. They reported that chronic diarrhea and extended family history by history of tuberculosis, rheumatic diseases and malignancy were statistically important in IEI predicting. They also suggested (but not evaluated) that non-infectious signs, such as immune dysregulation diseases and malignancies, should be considered as warning signs of IEI [24].

Due to the fact that many recent publications are focused on non-infectious phenotypes of IEI and given that other authors suggest the need for extending 10 warning signs of autoimmunity and malignancies [10,11], we evaluated four non-infectious signs in this study. The objective of this study was to determine and compare the utility of the JMF warning signs and the JMF warning signs extended by four non-infectious signs in identifying IEI.

## 2. Materials and Methods

The retrospective study included 2851 patients referred with suspected IEI to the Clinic at the Department of Pediatrics, Hematology and Oncology in the years 2003–2015.

58.8% were male and 41.2% were female. Of the 2851 enrolled patients, 2799 (98.17%) were under 18 years old. Only 52 (1.83%) adults were in the cohort. All patients were questioned about the 10 warning signs and 4 additional symptoms: severe eczema, allergies, hematologic and oncological disorders (malignancy and non- malignant: cytopenias, lymphoproliferation, hepatomegaly, splenomegaly), and autoimmunity (Table 2).

Enrolled patients were asked about parental consanguinity.

The retrospective chart included the following blood tests: the complete blood count, serum IgA IgM, IgG with IgG sub-classes level, serum complement C3, C4, and, if it was required, flow cytometry of T, B, and natural killer cells, Phagotest- FC, isohemagglutinin levels, vaccine response, and molecular analysis. The study excluded patients with a positive history of HIV infection/AIDS, immunosuppressive therapy, chemo- and radiotherapy, and any conditions with excessive loss of proteins. IEI were diagnosed according to the clinical European Society for Immunodeficiencies (ESID) diagnostic criteria. Genetic confirmation was necessary in some individuals.

IEI patients were distributed according to the International Union of Immunological Societies (IUIS) Primary Immunodeficiency Classification Committee [10,11]. Individuals with definable IEI not classified into any categories according to the IUIS were called “unclassified IEI”.

IEI patients were divided into 2 groups: with mild and severe immune defects. Mild IEI included immunodeficiencies, in which only infection prevention or symptomatic treatment of comorbidities is used, and in exceptional cases temporary substitution treatment with immunoglobulins is necessary (selected IgA deficiency, IgA deficiency, IgM deficiency, transient hypogammaglobulinemia of infancy, hypogammaglobulinemia, IgG subclass deficiency). Severe IEI included immune defects in which specific treatment is necessary, such as immunoglobulin substitution, biological therapies, immuno-suppressive therapy, hematopoietic cell transplantation (severe combined immunodeficiency, common variable immunodeficiency, agammaglobulinemia, chronic granulomatous disease, Wiskott-Aldrich syndrome, Nijmegen breakage syndrome, ataxia telangiectasia, autoimmune lymphoproliferative syndrome, DiGeorge syndrome, Shwachman Diamond syndrome, hemophagocytic lymphohistiocytosis, interferon-gamma receptor deficiency).

Sensitivity, specificity, positive and negative predictive values (PPV, NPV), and odds ratios (ORs) were calculated for the 10 and 14 warning signs.

### Statistical Analysis

Pearson’s chi-squared test, log-linear analysis, logistic regression, classification trees models were used in the statistical analysis.

To determine the limit values of variables, such as age and the number of symptoms, in the groups. The receiver operating characteristic curve (ROC) with the area under curve (AUC) calculating were capitalized. A *p*-value of less than 0.05 was considered statistically significant.

## 3. Results

### 3.1. Distribution of Inborn Errors of Immunity

**Figure 1:**
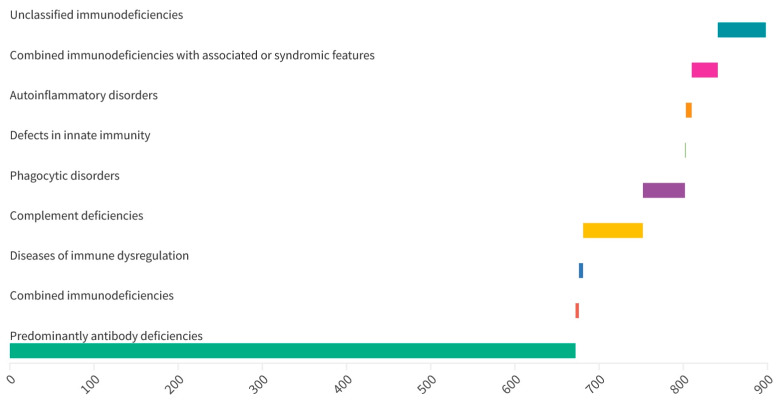
Distribution of inborn errors of immunity.

Of the 2851 enrolled patients, 896 (31.4%) were diagnosed with IEI, and in 1955 (68.6%) IEI was excluded. There were more patients with mild IEI than with severe IEI (822 vs. 74). Predominantly, antibody deficiencies represented the largest group of IEI in the cohort (Figure 1). Transient hypogammaglobulinemia of infancy (THI) was the most common IEI diagnosis followed by IgG hypogammaglobulinemia and IgA deficiency (Table 3).

### 3.2. Sex, Age and Consanguinity

**Figure 2:**
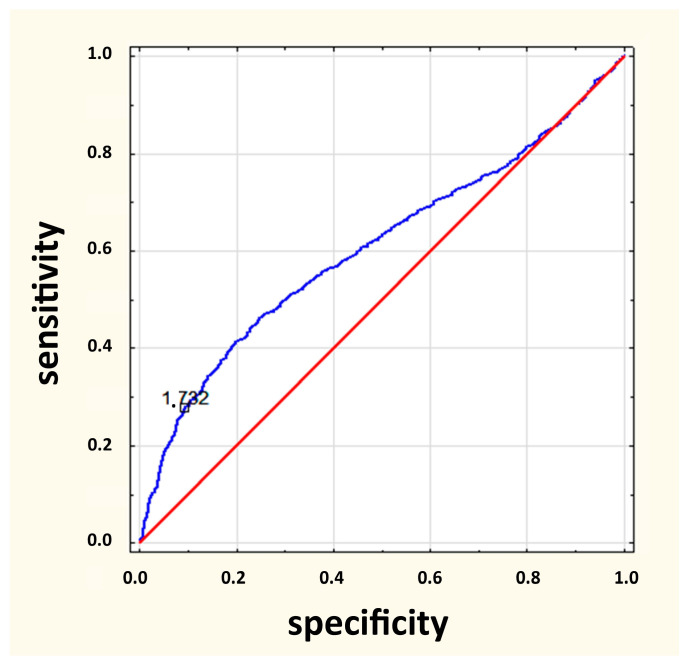
ROC analysis curve for age of patients.

In the IEI group, 552 (61.6%) were male and 344 (38.4%) were female. Further, 559 (62.4%) IEI patients were up to the age of five years. No parents were consanguineous. The analysis showed that at the time of reporting to the clinic, patients with IEI were significantly more often younger than those without IEI (*p* < 0.001). The ROC analysis indicated the age of 1.73 years as a possible cut-off point, which means that in the study group, children under one year and nine months of age were diagnosed with IEI more often than older children and young adults (Figure 2).

### 3.3. Frequency of the 14 Warning Signs in the Study Population

Only four symptoms occurred statistically significant more frequently in patients with IEI compared with patients without IEI. There were hematologic and oncological disorders, two or more deep-seated infections, a positive family history, and autoimmunity (Table 4).

Considering hematologic disorders and autoimmunity in IEI, cytopenias and juvenile idiopathic arthritis were the most common in this cohort, respectively (Table 5 and Table 6).

There were nine (1%) neoplasms in the subjects diagnosed with IEI, as shown in Table 7.

### 3.4. Diagnostic Value of the 14 Warning Signs in Predicting IEI

We reported an increased risk of IEI diagnosis when at least one warning sign of the following was present: hemato-oncological disorder, a positive family history, auto-immunization, or a minimum of two deep-seated infections including septicemia (Table 8).

Hemato-oncologic disorders and autoimmunity were the most significant predictors of IEI, with high specificity and PPV (98.7%/99.5% and 82%/77.5%, respectively, Table 9).

Comparing the group of patients with severe IEI (*n* = 74) and the group of people without IEI (*n* = 1955), the odds ratio of severe deficiencies in people meeting a given symptom from the list of 14 warning signs was calculated. The following symptoms significantly increased the chance of diagnosing severe IEI:

(1) haematological symptoms and neoplastic diseases (OR 89.26, 95% CI 48.18–165.34, *p* < 0.001)
(2) family history IEI (OR 25.24. 95% CI 12.84–49.58, *p* < 0.001)
(3) autoimmune diseases (OR 16.89, 95% CI 5.50–51.86, *p* < 0.001)
(4) inhibition of normal child development or weight gain (OR 6.16, 95% CI 3.60–10.52, *p* < 0.001)

An increased risk of severe IEI diagnosis occurs with the appearance of at least one warning sign, e.g., hemato-oncologic disorder, a positive family history, autoimmunity, or failure to thrive. Among them, the hemato-oncologic disorder has the highest PPV and sensitivity (Table 10).

Similarly, non-infectious symptoms were much stronger than any infectious warning sign in predicting humoral IEI (Table 11).

### 3.5. The List of 10 vs. 14 Warning Signs

For the analysis of diagnostic indicators of the lists of 10 and 14 warning symptoms, only those that statistically significantly increased the OR of IEI diagnosis were selected. They were:

(1) for a list of 10 symptoms when one symptom is met (OR = 1.15; *p* = 0.020)
(2) for a list of 14 symptoms when three symptoms are met (OR = 1.11; *p* = 0.020)

**Figure 3:**
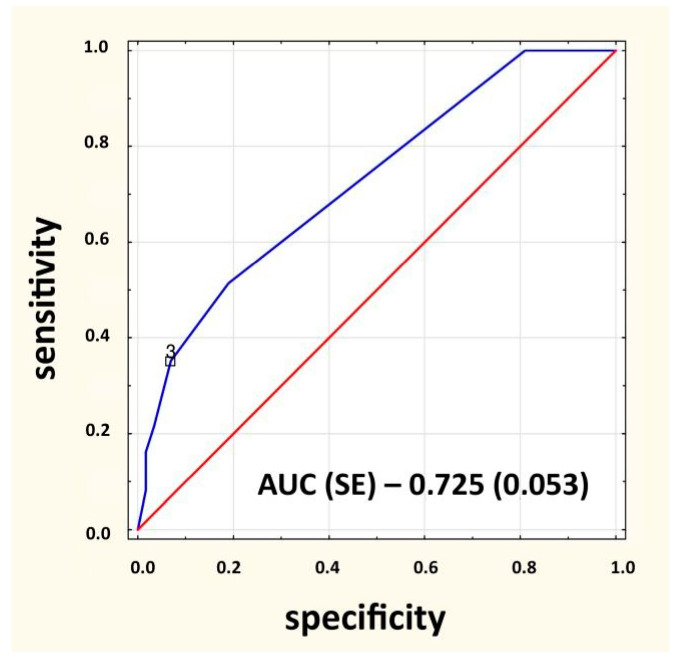
ROC analysis curve for the presence of three of 14 warning signs. AUC—area under the curve, SE—standard error.

The majority of IEI patients, constituting 65% (583 out of 896), met three symptoms from the list of 14 warning signs. More than half of them, 55.2% (322 out of 583) met only three symptoms from the list of the first 10 symptoms, 39% (227 out of 583) had two symptoms out of 10 symptoms and one additional symptom. Moreover 5.8% (34 out of 583) reported having one symptom from a list of 10 symptoms in combination with two additional symptoms. The mean number of 10 warning signs for subjects with mild and severe IEI was 2.0 (*p* = 0.270). The mean number of 14 warning signs in patients with severe and mild IEI were 3 and 2 respectively (*p* = 0.001). In addition, 183 people with IEI had no symptom from the list of 10 symptoms, which was 20% of the group of people with IEI (N = 896), and 126 people with IEI (14%) had no symptom from the list of 14 symptoms. A difference test between structure indices yielded *p* = 0.0007. Moreover, 15 people (20.3%) with severe disorders showed none of the 10 symptoms, and from the list of 14 symptoms, only five people (6.8%) showed none. A test of the difference between structure indicators yielded *p* = 0.0123. There were 20.4% and 14% of IEI patients without any signs from the 10 and 14 warnings signs, respectively (*p* < 0.001). Meanwhile, 20.3% and 6.8% of patients with severe IEI had no presence of any signs from the 10 and 14 signs, respectively (*p* = 0.012). It was found that the presence of one of 10 warning signs (OR = 1.15; *p* = 0.020) and three of 14 warning signs (OR = 1.11; *p* = 0.020) significantly increased the chance of identifying patients with IEI (Table 12 and Table 13, Figure 3).

## 4. Discussion

Eight out of ten symptoms from the JFM warning signs affect only infections [17]. In current study we have indicated that most infectious syndromes were not statistically more frequent in subjects with IEI compared with those without immunodeficiency. In this paper, only two symptoms from the JMF warning signs were statistically more frequent in patients with IEI. They were a family history, as shown in previous studies [18,25,26] and two or more deep-seated infections. A family history in a wide range was highlighted in a study conducted by Eldeniz et al. in Turkey [24]. A family history of tuberculosis, early sibling death, and parental consanguinity were evaluated in their study. The authors found that an extended family history of the signs was statistically significant in IEI predicting. The high rate of sibling death was also reported in the study by Reda et al., but it may be caused by the high rate (60%) of consanguineous parents [27]. Thus, it suggested that parental consanguinity and a family history of sibling deaths should be considered in countries where parental consanguinity is common [24]. No patients were questioned about a family history of tuberculosis and early sibling death in our cohort. Despite having no parental consanguinity in the study, and the fact that consanguineous marriages are extremely rare in Poland, further research would be worthwhile.

Chronic sinusitis did not correlate to the risk of IEI in our study. These results were influenced by the age structure in our cohort, where children younger than five years if age were dominant. Generally, sinusitis is a rare diagnosis in young children, because paranasal sinuses are not fully developed at that age. The diagnosis of CVID is most commonly made in adults [14]. Recurrent sinusitis was valuable in CVID prediction in our study, but there were only 2.1% of subjects with CVID, so it seems to have had no influence on the general results. In contrast, Joshi et al. [28] and Bjelac et al. [8] reported a noticeable association between recurrent sinusitis and IEI patients, but adults were the majority in their studies and there were many more subjects with CVID (14% and 77%, respectively) than in our cohort.

According to the literature, eczema affects 5–15% of patients with IEI [29,30], similarly to this study (20%), but in our cohort this symptom was not strong in predicting immune defects. An increased risk of cancer in IEI has been documented in many papers [31,32,33,34,35,36]. In this study, 1% of subjects with IEI suffered from neoplasms. In the literature, this value is estimated to range from 1% to 25% [1,37]. The huge range of observed values may result from the different ages of the observed populations. Because of the low number of malignancies in this study, we have not analyzed cancer as a single symptom in predicting IEI. According to the literature, patients with CVID are the vast majority of IEI patients with a cancer diagnosis. Vajdic et al. calculated that among IEI patients only subjects with CVID and ataxia teleangiectasia had an increased risk of malignancies [35]. Considering our paper, we speculate that relatively small group with CVID and ataxia teleangiectasia patients may be related to the low number of cancers in this research.

Interestingly, in predicting mild IEI, e.g., THI and sIgA, hematologic and oncologic symptoms were stronger than any infectious symptoms and the hemato-oncologic disorders were the only one that significantly increased the risk of the diagnosis of IgM deficiency. Further research is needed to establish whether malignancies correlate with a risk of antibody deficiency diagnosis excluding CVID. Our results showed a greater than 11-fold increased risk of the diagnosis of IEI when the hematologic and oncologic disorders were present. That association was much stronger in severe IEI (nearly 90-fold increased risk of the diagnosis of severe IEI). Interestingly, in predicting mild IEI, e.g., THI and sIgA, hematologic and oncologic symptoms were stronger than any infectious symptoms. The hemato-oncologic disorders represented the only factor that significantly increased risk of the diagnosis of IgM deficiency. Further research is needed to establish whether malignancies have a positive association with mild antibody deficiencies diagnosis.

We reported allergies in 12.5% of IEI patients, which was in accordance with Lugo-Reyes et al. [38]. Nevertheless, in our cohort, allergies were worthless in predicting IEI, similar to the work of Bjelac et al. [8].

In our research, autoimmunity was found only in 3.5% patients with IEI, less frequent than in the studies of others [39,40]. However, reporting of any autoimmunity was much stronger in predicting IEI than any reported infection. Because of the fact that cytopenia was a strong predictor of IEI in our research, we suggest that every patient with Evans syndrome (ES) should be evaluated for IEI. Many recent publications also emphasized the significance of identifying the underline immune dysregulation, such as primary immunodeficiencies in patients with ES, in which genetic investigations, such as next generation sequencing are needed and efficient [41,42].

In the current study, 20% of IEI patients had none of the symptoms from the JFM list. This finding was consistent with previous studies [18,25]. When comparing the 10 and 14 warning signs, there was a significantly lower number of IEI patients without any of the symptoms from the 14 signs. Similar to another study [43], we received higher specificity and NPV than sensitivity and PPV for the 10 and 14 warning signs. Conversely, Reda et al. have revealed that when at least two criteria of the JMF list were fulfilled, the sensitivity was 94% and the specificity was 64% [27]. Noticeably, in their study, severe IEI was dominant, and the rate of parental consanguinity was very high in the IEI group [27].

## 5. Conclusions

The JMF list modified with four additional signs has not improved its diagnostic sensitivity, specificity, PPV, and NPV. However, we documented that the 14 warning signs represent a better diagnostic tool than 10 warning signs in identifying patients with severe IEI. Raising awareness of non-infectious symptoms of IEI is needed in both the primary and specialist practice of non-immunologists, especially hematologists, oncologists, rheumatologists, gastroenterologists, and allergologists.

## Figures and Tables

**Table 1:** The 10 warning signs for pediatric primary immune deficiency.

	Sign
1.	≥4 new ear infections within 1 year
2.	≥2 serious sinus infections within 1 year
3.	≥2 months of oral antibiotic treatment with little effect
4.	≥2 episodes of pneumonia within 1 year
5.	Failure of an infant to gain weight or grow normally.
6.	Recurrent, deep skin or organ abscesses
7.	Persistent thrush in mouth or fungal infection on skin
8.	Need for intravenous antibiotics to clear infections
9.	≥2 deep-seated infections, including septicemia
10.	A family history of primary immunodeficiency

**Table 2:** Four additional warning signs of inborn errors of immunity.

	Sign
1.	Severe eczema
2.	Allergies
3.	Hematologic and oncologic disorders
4.	Autoimmunity

**Table 3:** Distribution of inborn errors of immunity.

Primary Immunodeficiency	Number	%
Transient hypogammaglobulinemia of infancy	189	21.1
IgG hypogammaglobulinemia	160	17.8
IgA deficiency	120	13.4
Selective IgA deficiency	88	9.8
C3, C4 complement deficiencies	67	7.5
IgM deficiency	63	7.0
Unclassified primary immunodeficiencies	57	6.4
IgG subclass deficiency	43	4.8
Common variable immunodeficiency disorders	19	2.1
DiGeorge syndrome	15	1.7
IgG subclass deficiency associated with IgA deficiency	14	1.6
IgA and IgM deficiency	8	0.9
Autoinflammatory diseases	7	0.8
Wiskott-Aldrich syndrome	6	0.7
Nijmegen breakage syndrome	5	0.6
Ataxia telangiectasia	5	0.6
CD19+ deficiency	5	0.6
Severe combined immunodeficiency	4	0.4
Shwachman-Diamond syndrome	4	0.4
Agammaglobulinemia	3	0.3
Autoimmune lymphoproliferative syndrome	3	0.3
Hemophagocytic lymphohistiocytosis	2	0.2
Chronic granulomatous disease	2	0.2
Severe congenital neutropenia	2	0.2
IgG and IgM deficiency	2	0.2
Interferon-gamma receptor deficiency	1	0.1

**Table 4:** Frequency of each warning sign in subjects with IEI compared with non IEI.

Warning Sign	IEI *n* (%): 896 (31.4%)	No IEI *n* (%): 1955 (68.6%)	*p*-Value
≥4 new ear infections within 1 year	607 (68%)	1575 (81%)	<0.001
≥2 serious sinus infections within 1 year	55 (6%)	195 (10%)	<0.001
≥2 months of oral antibiotic treatment with little effect	135 (15%)	290 (15%)	0.871
≥2 episodes of pneumonia within 1 year	281 (31.4%)	669 (34%)	0.132
Failure of an infant to gain weight or grow normally.	81 (9.0%)	138 (7%)	0.065
Recurrent, deep skin or organ abscesses	14 (2%)	56 (3%)	0.037
Persistent thrush in mouth or fungal infection on skin	101 (11%)	276 (14%)	0.037
Need for intravenous antibiotics to clear infections	303 (33.8%)	631 (32%)	0.415
≥2 deep-seated infections, including septicemia	49 (5%)	61 (3%)	0.002
A family history of primary immunodeficiency	41 (5%)	25 (1%)	<0.001
Severe eczema ^1^	180 (20%)	429 (22%)	0.262
Allergies ^1^	112 (12.5%)	287 (15%)	0.119
Hematologic and oncologic disorders ^1^	114 (13%)	25 (1%)	<0.001
Autoimmunity ^1^	31 (3.5%)	9 (0.5%)	<0.001

^1^ 4 non-infectious warning signs added to 10 warning signs of IEI. Explanation of abbreviation—IEI, inborn errors of immunity.

**Table 5:** Hematologic disorders in IEI patients.

Hematologic Disorders	IEI (*n*)
Cytopenias	CVID (19) DiGerorge syndrome (8) Hipogamm. (8) sIgAD (6) IgMD (6) WAS (6) AT (5) NBS (5) SCID (4) Shwachman-Diamond syndrome (3) HLH (2)
Non-malignant hepatomegaly	SCID (4) CGD (2) ALPS (1) AT (2) CVID (1) IgMD (1)
Non-malignant splenomegaly	ALPS (3) HLH (2) SCID (4)
Non-malignant lymphadenopathy	CVID (8) ALPS (2) CGD (2)
Total number of hematologic disorders	105

Explanation of abbreviations—IEI, inborn errors of immunity; CVID, common variable immunodeficiency disorders, sIgAD, selective IgA deficiency; IgMD, IgM deficiency; WAS, Wiskott-Aldrich syndrome; AT, ataxia-teleangiectasia; NBS, Nijmegen breakage syndrome; SCID, severe combined immunodeficiency; ALPS, autoimmune lymphoproliferative syndrome; CGD, chronic granulomatous disease; HLH, Hemophagocytic lymphohistiocytosis.

**Table 6:** Autoimmune diseases in IEI patients.

Autoimmunity	IEI (*n*)
Juvenile rheumatoid arthritis	sIgAD (6) IgAD (2) Hipogamm. (2) IgMD (1)
Hashimoto disease	sIgAD (3) Subclass deficiency (2) CVID (1)
Diabetes mellitus type 1	sIgAD (2) Hipogamm. (1)
Immune thrombocytopenia	CVID (1) Hipogamm. (1) ALPS (1)
Ulcerative colitis	sIgAD (2) CVID (1)
Celiac disease	sIgAD (2)
Psoriasis	THI (1)
Autoimmune hemolytic anemia	THI (1) ALPS (1)
Total number of autoimmune disorders	31

Explanation of abbreviations—IEI, inborn errors of immunity; CVID, common variable immunodeficiency disorders, sIgAD, selective IgA deficiency; IgMD, IgM deficiency; ALPS, autoimmune lymphoprolifeartive syndrome; THI, transient hypogammaglobulinemia of infancy.

**Table 7:** Cancers in IEI patients.

IEI	Cancer	Number of Cancers
THI	Wilms tumor	1
	Plexiform neurofibroma	2
CVID	Seminoma testis	1
	Pulmonary cancer	1
Hypogammaglobulinemia	AML	2
NBS	T-ALL	2
Total number of cancers		9

Explanation of abbreviations—IEI, inborn errors of immunity; THI, transient hypogammaglobulinemia of infancy; CVID, common variable immunodeficiency disorders, NBS, Nijmegen breakage syndrome; AML, acute myeloid leukemia; T-ALL, T-lymphoblastic leukemia.

**Table 8:** Odds ratio for the 14 warning signs in subjects with IEI compared with non IEI.

Warning Sign	Odds Ratio	*p*-Value	95% Confidence Interval
≥4 new ear infections within 1 year	0.50	<0.001	0.42–0.60
≥2 serious sinus infections within 1 year	0.59	<0.001	0.43–0.80
≥2 months of oral antibiotic treatment with little effect	1.01	0.871	0.81–1.27
≥2 episodes of pneumonia within 1 year	0.87	0.132	0.74–1.04
Failure of an infant to gain weight or grow normally	1.30	0.065	0.98–1.73
Recurrent, deep skin or organ abscesses	0.53	0.037	0.29–0.97
Persistent thrush in mouth or fungal infection on skin	0.77	0.037	0.60–0.98
Need for intravenous antibiotics to clear infections	1.07	0.415	0.90–1.26
≥2 deep-seated infections, including septicemia	1.79	0.002	1.22–2.63
A family history of primary immunodeficiency	3.70	<0.001	2.23–6.12
Severe eczema ^1^	0.89	0.262	0.73–1.08
Allergies ^1^	0.83	0.119	0.65–1.05
Hematologic and oncologic disorders ^1^	11.25	<0.001	7.23–17.50
Autoimmunity ^1^	7.74	<0.001	3.66–16.36

^1^ 4 non-infectious warning signs added to 10 warning signs of IEI.

**Table 9:** Sensitivity, specificity, positive predictive value, and negative predictive value for the 14 warning signs in subjects with IEI compared with non IEI.

Warning Sign	Sensitivity (%)	Specificity (%)	PPV (%)	NPV (%)
≥4 new ear infections within 1 year	67.7	19.4	27.8	56.8
≥2 serious sinus infections within 1 year	6.1	90.0	22.0	67.7
≥2 months of oral antibiotic treatment with little effect	15.1	85.2	31.8	68.6
≥2 episodes of pneumonia within 1 year	31.4	65.8	29.6	67.6
Failure of an infant to gain weight or grow normally	9.0	92.9	37.0	69.0
Recurrent, deep skin or organ abscesses	1.6	97.1	20.0	68.3
Persistent thrush in mouth or fungal infection on skin	11.3	85.9	26.8	67.9
Need for intravenous antibiotics to clear infections	33.8	67.7	32.4	69.1
≥2 deep-seated infections, including septicemia	5.5	96.9	44.5	69.1
A family history of primary immunodeficiency	4.6	98.7	62.1	69.3
Severe eczema ^1^	20.1	78.1	29.6	68.1
Allergies ^1^	12.5	85.3	28.1	68.0
Hematologic and oncologic disorders ^1^	12.7	98.7	82.0	71.2
Autoimmunity ^1^	3.5	99.5	77.5	69.2

^1^ 4 non-infectious warning signs added to 10 warning signs of IEI; Explanation of abbreviations—PPV, positive predictive value; NPV, negative predictive value.

**Table 10:** Warning signs that significantly increased risk of a diagnosis of severe IEI comparing to subjects without IEI (*p* < 0.05).

Warning Sign	Sensitivity (%)	Specificity (%)	PPV (%)	NPV (%)	OR
Failure of an infant to gain weight or grow normally	31.9	92.9	13.8	97.5	6.16
A family history of primary immunodeficiency	24.6	98.7	40.5	97.4	25.24
Hematologic and oncologic disorders ^1^	53.6	98.7	59.7	98.4	89.26
Autoimmunity ^1^	7.2	99.5	35.7	96.8	16.89

^1^ Non-infectious warning signs added to 10 warning signs of IEI; Explanation of abbreviations—PPV, positive predictive value; NPV, negative predictive value; OR, odds ratio.

**Table 11:** Odds ratio for the recognition of selected IEI.

Warning Sign	Hypogammaglobulinemia *n* (%): 138 (15.4)	Transient Hypogammaglobulinemia of Infancy *n* (%): 189 (21.1)	IgM Deficiency *n* (%): 63 (7.0)	Selective IgA Deficiency *n* (%): 88 (9.8)	CVID *n* (%): 19 (2.1)
≥4 new ear infections within 1 year	0.55 **	0.30 **	0.62	0.57*	1.20
≥2 serious sinus infections within 1 year	0.48	0.09 **	0.21	0.31	3.22 *
≥2 months of oral antibiotic treatment with little effect	1.14	0.83 **	0.09*	0.99	3.34 *
≥2 episodes of pneumonia within 1 year	1.47 *	0.71 *	1.00	0.76	2.00
Failure of an infant to gain weight or grow normally	1.36	1.21	0.80	0.62	0.50
Recurrent, deep skin or organ abscesses	0.49	0.54	0.30	0.39	0.30
Persistent thrush in mouth or fungal infection on skin	0.74	0.64	0.20	1.15	0.50
Need for intravenous antibiotics to clear infections	1.08	1.71 **	1.2 *	0.61	2.07
≥2 deep-seated infections, including septicemia	1.65	2.29 **	0.8	1.09	1.01
A family history of primary immunodeficiency	6.03 **	1.66	0.89	2.72	9.08 *
Severe eczema ^1^	1.16	0.56 **	1.05	1.18	0.55
Allergies ^1^	0.70	0.57 *	1.70	0.74	1.63
Hematologic and oncologic disorders ^1^	8.71 **	6.17 **	6.65 **	4.65 **	45.03 **
Autoimmunity ^1^	4.80 *	1.15	1.20	30.88 **	25.44 **

*—*p* < 0.05; **—*p* < 0.001; Explanation of abbreviations—CVID, common variable immunodeficiency. ^1^—four new symptoms in addition to the existing list of 10 warning signs.

**Table 12:** Prediction of identifying patients with IEI by presence of 1, 2 or 3 of the 10 warning signs.

**Diagnostic Parameter**	**1 of 10**	**2 of 10**	**3 of 10**
Sensitivity (%)	27.0	24.2	18.0
Specificity (%)	75.8	72.5	80.2
Odds ratio	1.15	0.84	0.88
Positive predictive value (%)	32.2	28.8	29.4
Negative predictive value (%)	70.8	67.6	68.1

**Table 13:** Prediction of identifying patients with IEI by presence of 1, 2 or 3 of the 14 warning signs.

**Diagnostic Parameter**	**1 of 14**	**2 of 14**	**3 of 14**
Sensitivity (%)	19.6	23.4	20.6
Specificity (%)	79.6	73.8	79.6
Odds ratio	0.95	0.86	1.11
Positive predictive value (%)	30.7	29.1	31.5
Negative predictive value (%)	68.4	67.8	68.8

## Data Availability

Not applicable.
